# A multi-center, randomized, parallel-group study to compare the efficacy of enhanced cognitive behavior therapy (CBT-E) with treatment as usual (TAU) for anorexia nervosa: study protocol

**DOI:** 10.1186/s13030-023-00277-2

**Published:** 2023-05-29

**Authors:** Nobuhiro Nohara, Yukari Yamanaka, Mikiko Matsuoka, Tadahiro Yamazaki, Keisuke Kawai, Shu Takakura, Nobuyuki Sudo, Tetsuya Ando, Yutaka Matsuyama, Susan Byrne, Riccardo Dalle Grave, Zafra Cooper, Kazuhiro Yoshiuchi

**Affiliations:** 1 Department of Stress Sciences and Psychosomatic Medicine, Graduate School of Medicine, The University of Tokyo, 7-3-1, Hongo, Bunkyo-ku, Tokyo, 113-8655 Japan; 2 Department of Psychosomatic Medicine, Kohnodai Hospital, National Center for Global Health Medicine, 1-7-1 Kohonodai, Ichikawa City, 272-8516 Chiba Japan; 3 Department of Psychosomatic Medicine, Kyushu University Hospital, 3-1-1 Maidashi, Higashi-ku, Fukuoka, 812-8582 Japan; 4 Department of Psychosomatic Medicine, Graduate School of Medical Sciences, Kyushu University, 3- 1-1 Maidashi, Higashi-ku, Fukuoka, 812-8582 Japan; 5 Department of Psychosomatic Internal Medicine, International University of Health and Welfare Narita Hospital, 852 Hatakeda, Narita City, 286-8520 Chiba Japan; 6 Department of Biostatistics, School of Public Health, Graduate School of Medicine, The University of Tokyo, 7-3-1, Hongo, Bunkyo-ku, Tokyo, 113-8655 Japan; 7 SWAN Centre, Perth and School of Psychology, University of Western Australia, Perth, Australia; 8 Department of Eating and Weight Disorders, Villa Garda Hospital, Via Monte Baldo, 89, Verona, Garda, 37016 Italy; 9 Department of Psychiatry, Yale School of Medicine, New Haven, USA

**Keywords:** Adolescent, Adult, Anorexia nervosa, Feeding and eating disorders, Psychological treatments, Randomized controlled trial, Enhanced cognitive behavioral therapy

## Abstract

**Background:**

The superiority of Enhanced Cognitive Behavior Therapy (CBT-E) with regard to weight gain and improvement of psychopathology of eating disorders for patients with anorexia nervosa (AN) over other psychotherapies and treatment as usual (TAU) has not been demonstrated in randomized controlled trials (RCTs). However, a previous RCT showed that patients with AN whose baseline body mass index (BMI) was less than 17.5 kg/m^2^ gained more weight when treated with CBT-E than with other psychotherapies. The aim of the study is to compare the efficacy of CBT-E and TAU for patients with AN. It was hypothesized that CBT-E would be superior to TAU, at least in terms of weight gain, as most patients with AN are likely to have a BMI lower than 17.5 kg/m^2^.

**Methods/design:**

A randomized parallel-group multicenter trial will be conducted in three teaching hospitals in Japan between January 2023 and March 2026. Patients with DSM-5 AN, aged 16 years and older, with a BMI equal to or above 14.0 and below 18.5 will be eligible to participate. 56 patients will be randomly and evenly assigned to two intervention groups (CBT-E and TAU). Those assigned to CBT-E will be offered 25–40 sessions in accordance with their initial BMI. Patients assigned to TAU will have at least one session every 2 weeks, with the number of sessions and treatment period not fixed in advance. The primary outcome is BMI at 40 weeks after treatment initiation. The secondary outcomes are the results from the Japanese version of the Eating Disorder Examination Questionnaire and Clinical Impairment Assessment questionnaire to measure eating disorder psychopathology and psychological impairment. The follow-up assessment will be performed 6 months after the 40-week assessment.

**Discussion:**

This multi-center randomized controlled study will probably evaluate the efficacy of CBT-E compared with TAU for patients with more severe AN than in previous studies since Japanese patients are likely to have a lower BMI than those in Western countries. While it may be difficult to generalize the results of a study conducted in Japan, it would be valuable to clarify the efficacy of CBT-E as a treatment package.

**Trial registration:**

UMIN, UMIN000048847. Registered 12 Sep 2022.

**Supplementary Information:**

The online version contains supplementary material available at 10.1186/s13030-023-00277-2.

## Background

Anorexia nervosa (AN) is a serious psychiatric disorder characterized by intense fear of weight gain, abnormal eating, and disturbed body image and body weight [1]. Psychotherapeutic interventions are the first-line treatment. Several treatment guidelines, such as the Clinical Practice Guidelines of the Royal Australian and New Zealand College of Psychiatrists, the Dutch Practice Guideline for the Treatment of Eating Disorders, and the UK National Institute for Health and Care Excellence Clinical Guidelines for Eating Disorders (NICE guidelines) recommend eating disorder focused cognitive behavior therapy (CBT) for eating disorders (EDs). Enhanced Cognitive Behavior Therapy (CBT-E) is the form of CBT with the strongest evidence base and, as such, is a, treatment of choice for AN [2–4]. Nevertheless, only limited research has provided support for CBT-E for adult and adolescent patients with AN.

For patients with bulimia nervosa (BN) or binge eating disorder (BED), evidence that CBT-E is more effective in terms of improving alleviating eating disorder features and general psychopathology than other treatments has been accumulating [5–8].

For patients with AN, cohort studies have shown that CBT-E is effective both for adult and adolescent patients with AN. A cohort study of adult patients with AN demonstrated a substantial increase in weight and marked improvement in ED symptoms in those patients who completed outpatient CBT-E treatment [9]. In adolescent patients with AN receiving outpatient CBT-E, eating disorder and general psychopathology improved to a similar degree as with adult patients by the end of treatment with both considerable weight gain and reduced scores for clinical impairment, eating disorder, and general psychopathology at the end of treatment and also at 20-week follow-up [10, 11] .

Network meta-analysis suggests that CBT-E may be superior to both the Maudsley Anorexia Nervosa Treatment for Adults (MANTRA) and Specialist Supportive Clinical Management (SSCM) recommended by the NICE guideline [4] in terms of weight restoration, though confidence in the evidence was low due to heterogeneity or inconsistency between studies [12].

Despite these positive findings favoring CBT-E, some studies have failed to find that it is superior to other active psychological treatments. The Anorexia Nervosa Treatment of OutPatient (ANTOP) study compared the efficacy of two manual-based outpatient treatments for AN: focal psychodynamic therapy, CBT-E, and treatment as usual (TAU) and showed that there were no significant differences in weight gain between any of these treatments [13]. The Strong Without Anorexia Nervosa (SWAN) study, an RCT that compared three psychological treatments for AN (MANTRA, SSCM, and CBT-E) in outpatient settings also failed to show that there were significant differences in weight regain, improvement in ED psychopathology, and psychosocial impairments between these treatments [14].

Although the superiority of CBT-E over other therapies has not been demonstrated in RCTs, subgroup analysis of patients with a baseline BMI less than 17.5 kg/m^2^ in the ANTOP study revealed that mean BMI at the end of treatment was significantly higher in those who received CBT-E than in those who received focal psychodynamic therapy [13]. A similar finding was obtained in the previously mentioned cohort study [9]. These results suggest that the CBT-E may differentially be effective in those with AN and a low BMI. In Japan, most patients diagnosed with AN have a BMI lower than 17.5 kg/m^2,^ and therefore the study might provide the opportunity to investigate the possibility that CBT-E is more efficacious for patients with AN and with lower BMIs than other active treatments both in terms of weight gain and improvement in ED psychopathology. Previous RCTs have included patients with AN diagnosed by DSM-IV criteria, while this study will employ DSM-5 criteria, providing the opportunity to investigate outcome in this group.

## Objectives and hypotheses

The aim of this study is to compare the efficacy of CBT-E with TAU for patients with AN in a RCT. It is hypothesized that CBT-E will be superior to TAU, in terms of weight gain, especially in Japan as most patients with AN are likely to have a BMI less than 17.5 kg/m^2^.

## Methods

### Trial design

**Fig. 1:**
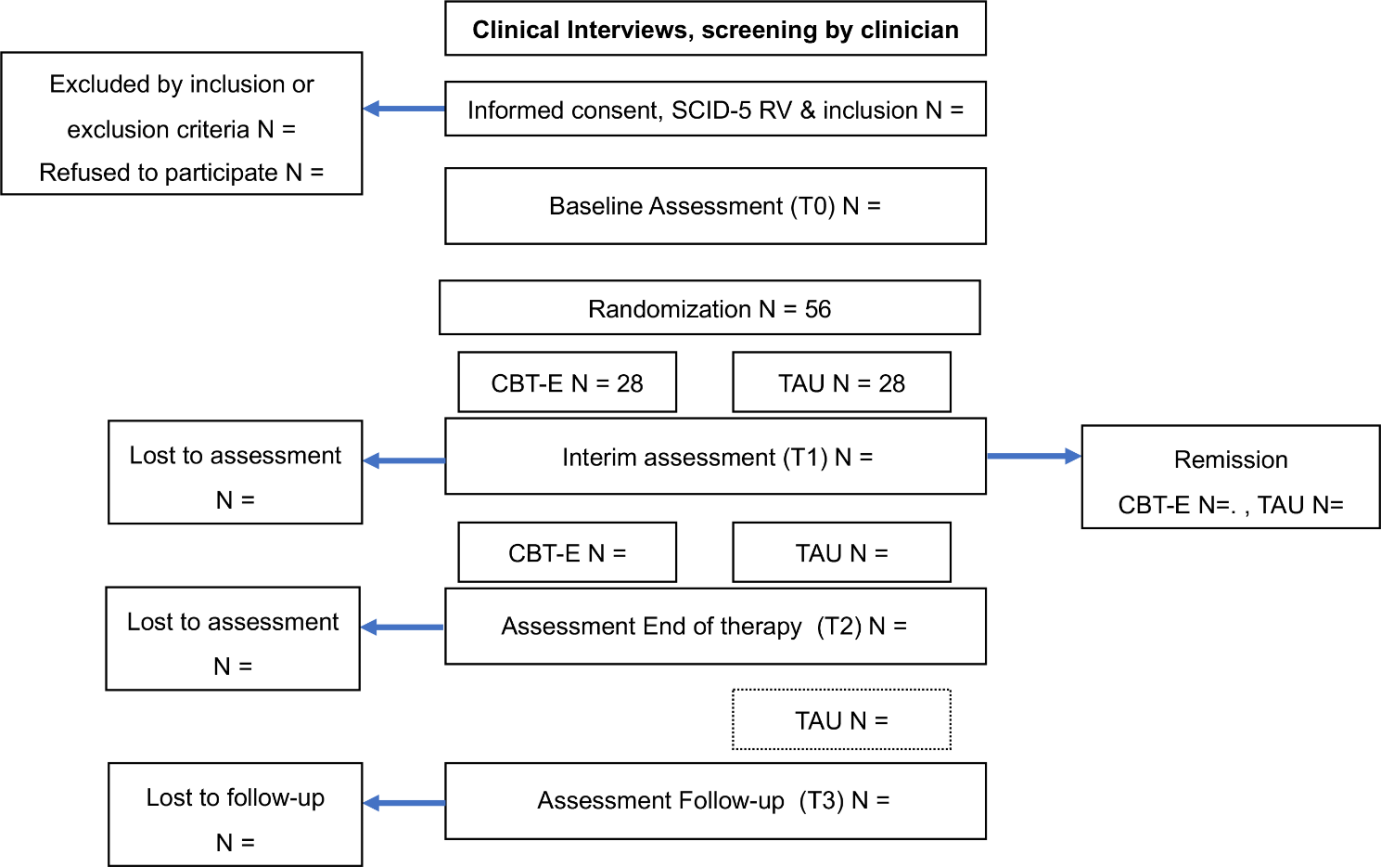
Flow diagram of the participants. *CBT-E*: Enhanced cognitive behavioral therapy, *TAU*: treatment as usual. *T0*: baseline, *T1*: week 20, *T2*: end of treatment, week 40, *T3*: follow-up, 6 months after *T3*. *SCID-5 RV*: Structured Clinical Interview for DSM-5 Disorders, Research Version, *EDE-Q*: The Eating Disorder Examination Questionnaire, *CIA*: Clinical Impairment Assessment questionnaire. The number and frequency of sessions of CBT-E vary according to baseline BMI shown in the Supplementary Table S1. The treatment period and intensity of the TAU are not specifically determined in advance. When participants are remitted at the interim assessment (*T1*), the study treatment will be terminated. Remission was defined as BMI ≥ 18.5 and the EDE-Q-J global score below 1 stand deviation above the community mean

This is a multicenter study, stratified by trial sites (The University of Tokyo Hospital, Kohnodai Hospital, and Kyushu University Hospital) and BMI (below 16.0, above or equal to 16.0 and below or equal to 17.5, and above 17.5 and below 18.5), using dynamic balanced randomization and a no-blind, parallel-group design that will be conducted in Japan [15]. Figure 1 shows the flow diagram of participants through the trial.

### Participants

The present study will recruit 56 participants. The University of Tokyo Hospital will recruit 23 patients, Kohnodai Hospital 10 patients, and Kyushu University Hospital 23 patients. The participants will be recruited via online advertisements through official websites and advertisements on hospital bulletin boards. Patients referred from another medical facility with suspected AN will also be recruited at their first visit. To be eligible to participate in this study, participants must meet all the inclusion and exclusion criteria listed in Table 1.

**Table 1:** Eligibility criteria for participants

Inclusion criteria	• Those who meet the criteria of DSM-5 for AN. • Those who are 16 years old or older when they give consent to participate in the study. • Those whose BMI are equal to or above 14.0 and below 18.5. • Those living in Japan who are literate in Japanese. • Those who understand the study concepts and give written informed consent.
Exclusion criteria	• Those who are receiving or have received any type of structured psychotherapies including CBT-E, Family therapy, Maudsley Model of Anorexia Nervosa Treatment for Adults (MANTRA), Specialist Supportive Clinical Management (SSCM), Interpersonal Therapy (IPT) within the last year. • Those who have mental illnesses* including schizophrenia, bipolar disorder or substance use disorder, or somatic disease that impedes the study treatment. • Those who are currently receiving antipsychotic medication except for antidepressants, anxiolytics, or hypnotics (The participants on antidepressants or hypnotics will not be excluded as far as they are on stable or reducing dose during the study). • Those who have an intellectual disability. • Those who have suicidal ideation. • Those who are pregnant or might become pregnant. • Those who have difficulty in attending therapy on schedule. • Those whom therapists or physicians consider as ineligible to participate in this study.

*: The Structured Clinical Interview for DSM-5, Research Version (SCID-5-RV), is used to exclude patients with any history of schizophrenia, bipolar disorder, or substance use disorder

### Settings and locations

The study will be carried out at The University of Tokyo Hospital, Kohnodai Hospital, National Center for Global Health and Medicine, and Kyushu University Hospital between 2023 and 2026. The University of Tokyo Hospital is one of the largest tertiary care teaching hospitals with more than 1,200 beds located in Tokyo, the political and economic center of Japan, with a population of approximately 14 million. Kohnodai Hospital is a secondary care hospital with approximately 300 beds in Ichikawa, a city located within 10 to 20 km from the center of Tokyo, with a population of approximately 500,000. Kyushu University Hospital is also one of the largest tertiary care teaching hospitals with more than 1,200 beds located in Fukuoka, the sixth-largest city in Japan, with a population of approximately 1.6 million.

### Interventions

Participants will be randomly assigned to receive either CBT-E or TAU. They will not be allowed to receive any other psychotherapy except the treatment they are assigned during the treatment and follow-up period. CBT-E and TAU will be provided in outpatient settings by different therapists, who have had more than 2 years of experience in treating patients with EDs. The assignment of therapists will be carried out in a manner that there is not substantial difference in years of experience in treating EDs between the two treatment therapists. CBT-E will be offered only by physicians or psychotherapists who have been supervised or are currently being supervised by Prof. Zafra Cooper, who is one of the most prominent international CBT-E specialists. When the treatment sessions are conducted by psychotherapists, the attending physician monitors the participants’ physical conditions before or after each session. If their BMI falls below 14 or any medical instability emerges, treatment will be suspended for up to 3 weeks for inpatient treatment or corresponding treatment to address the condition. The BMI of 14 was considered the lower cutoff limit in previous studies and in the guidelines of Japan Society for Eating Disorders [13, 14, 16]. If the patient does not recover within 3 weeks, they will be withdrawn from the study. If participants are remitted at 20 weeks after treatment initiation, the treatment will be terminated. Remission will be defined with reference to previous studies: a BMI ≥ 18.5 and an EDE-Q-J global score less than 1 SD above the community mean [6, 8, 17, 18].

#### CBT-E

CBT-E is a treatment for the full range of ED presentations, irrespective of ED diagnosis. With patients who are underweight, CBT-E has three phases [19]. In the first phase, the emphasis is on increasing patients’ motivation to change and helping them recognize their current maladaptive thinking and behaviors and the processes maintaining them. Initially, patients are encouraged to commence and maintain self-monitoring (one of the crucial factors in CBT-E), adopt a regular pattern of eating, and actively make the decision to address weight restoration. Subsequently the treatment is focused on continuing to address weight restoration and eating disorder psychopathology, such as extreme concerns about their shape and weight. Patients are encouraged to gain weight until their BMI falls within the normal range (18.5–24.9 kg/m^2^). In the final phase, the focus is on helping patients maintain the changes that they have achieved and developing strategies in advance for risky situations that might trigger setbacks. Patients assigned to CBT-E are offered 25–40 sessions that last about 50 min over a 40-week period. The focused form of CBT-E will be offered and the number of sessions varies according to the participants’ BMI at their first visit (below 16: 40 sessions, above or equal to 16 and below or equal to 17.5: 30 sessions, above 17.5 and below 18.5: 25 sessions) to allow for enough time to regain weight, following the protocol devised for the SWAN study [14]. The detailed information about the schedule and frequency of sessions is shown in the Supplementary Table S1.

#### TAU

TAU consists of establishing a therapeutic relationship, diagnosis and assessment, psychoeducation, nutritional guidance, food records, behavioral techniques such as stimulus control, and supportive psychotherapy. TAU does not employ any treatment component or strategy of structured psychotherapies, such as CBT-E or IPT.

The total number and frequency of sessions provided for the patients and when to use treatment modules are not specifically determined in advance to be approximately congruent with the treatments provided in usual outpatient settings. Patients assigned to TAU have at least one session every 2 weeks that lasts 15–30 min. The duration of treatment can be longer or shorter than 40 weeks according to the patients’ status.

### Outcomes

**Fig. 2:**
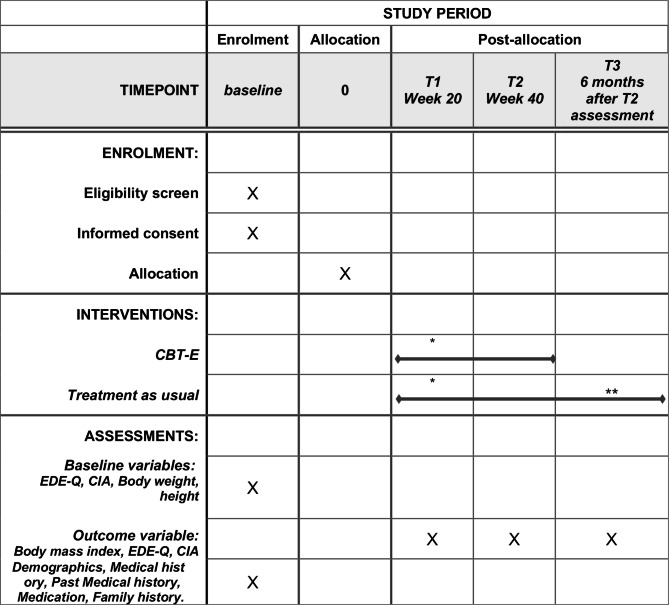
The schedule of enrollment, interventions, and assessments. *: When participants are remitted at 20 weeks (T1 assessment), the treatment will be terminated. However, T2 and T3 assessment will be undertaken for those remitted at T1 assessment **: The duration and frequency of treatment as usual vary according to the participants’ conditions

The primary outcome is BMI (kg/m^2^) at 40 weeks after treatment initiation. The secondary outcomes are eating disorder symptoms/psychopathology as measured by the Japanese versions of the EDE-Q (EDE-Q-J) [20] and impairment measured by the CIA [21]. Measurements will be performed at four time points: at baseline (before randomization), at 20 weeks, at 40 weeks after treatment initiation, and at 6 months follow-up after the assessment at 40 weeks. Figure 2 shows the schedule of enrollment, interventions, and assessments.

### The Japanese version of eating disorder examination questionnaire (EDE-Q-J)

The Eating Disorder Examination Questionnaire (EDE-Q) is a widely used self-report questionnaire that assesses the core psychopathology of EDs and has been used to assess the effectiveness of psychotherapy for patients with EDs [18, 22, 23]. The EDE-Q was adapted from the interview-based Eating Disorder Examination (EDE 16.0). The structure of the Japanese version of EDE-Q (EDE-Q-J) is not consistent with that of original EDE-Q. However, EDE-Q-J has sufficient reliability and validity, and three subscales of EDE-Q-J are interpretable [20].

### The Japanese version of clinical impairment assessment questionnaire (CIA)

The original version of CIA was developed as a self-report questionnaire to measure the nature and severity of psychosocial impairment resulting from ED psychopathology [21]. The Japanese version of CIA has been demonstrated to be reliable and validated as with the original version [24].

### Sample size

To detect an effect size of 0.96 in BMI increase estimated in previous studies, with a two-sided 5% significance level and power of 80%, a sample size of 28 patients per group was deemed necessary, given an anticipated drop rate of 30% [13, 14]. A 24-month enrollment period is needed to recruit this number of patients. The number of participants recruited at each site is as follows: The University of Tokyo Hospital will recruit 23 patients, Kohnodai Hospital 10 patients, and Kyushu University Hospital 23 patients.

### Randomization

The generation and implementation of the centralized randomization sequence will be conducted independently from the trial teams using the cloud version of UMIN Internet Data and Information system for Clinical and Epidemiological research (cloud INDICE). Participants are randomly assigned to one of the two trial arms in a 1:1 ratio, using a computer-generated, dynamic procedure (minimization) stratified by trial sites and BMI (below 16, above or equal to 16 and below or equal to 17.5, and above 17.5 and below 18.5). Participants will be allocated, with an 80% probability, to the group that minimizes between-group differences in BMI and trial site among all participants. Whereas patients, psychotherapists, and physicians are aware of which group they belong to, outcome assessors and data analysts will be kept blinded to the allocation.

### Statistical methods

The primary outcome is a BMI at 40 weeks after treatment in the intention-to-treat population. The secondary outcomes are eating disorder psychopathology and impairment measured by EDE-Q-J and CIA questionnaires. Multilevel modeling will be applied to longitudinal analyses of BMI and scores on the EDE-Q-J and the CIA of patients nested within hospitals.

### Ethics and dissemination

Ethical approval for the study has been obtained from the central review board of the ethics committees of the Graduate School of Medicine of the University of Tokyo (2021349NI). Participants are not exposed to risk and receive verbal and written information before enrolling in the study. All participants will give their informed consent for participation in this study. The study will be conducted according to the principles of the Declaration of Helsinki.

The results of the trial will be disseminated via peer-reviewed publications and presentations at relevant conferences. This trial is registered with the UMIN Clinical Trials Registry as UMIN000048847.

## Discussion

The aim of the present study is to provide new evidence regarding the efficacy of CBT-E for adolescents and adult patients with AN in outpatient settings. Previous randomized controlled trials have failed to demonstrate the superiority of CBT-E over other psychotherapeutic treatments both in regard to weight recovery and reducing ED psychopathology [13, 14]. However, there is preliminary evidence that in those with a lower relative BMI, CBT-E may be superior to other active treatments in terms of weight restoration, with patients achieving a higher BMI at the end of treatment [9, 13]. The distribution of BMI of the patients with AN in this study will be skewed toward lower BMIs as compared to that of the previous studies based on Western populations because the mean BMI in Japan is lower than that in Western countries [25], and the mean BMI of patients with AN restricting type (AN-R) in Japan has been decreasing over the past 30 years from 14.0 to 13.4 kg/m^2^ [26]. If this finding is robust, the present study will be expected to provide evidence of the superiority of CBT-E over TAU at least in terms of weight recovery.

The study will have several strengths. First, the study will include those with more severe AN with a lower BMI range than previous RCTs. The ANTOP study had a BMI limit of 15 and above, and the SWAN study included patients with an average BMI of 16.59 (S.D. = 1.35). The present study will include those with a BMI of 14 and above from the population with a lower BMI range than previous studies, which is likely to result in the inclusion of patients with more severe AN than previous studies.

Second, the study will be likely to include more adolescents than previous RCTs and might make it possible to evaluate the efficacy of CBT-E in younger patients in RCTs. While CBT-E for adolescents has previously shown efficacy in achieving weight gain and improving ED psychopathology in younger patients [10, 11], these studies had no control or comparison treatment.

Third, the treatments will be delivered in ‘real-world’ outpatient settings by therapists with varying degrees of experience. For this reason, the content of TAU is not strictly prescribed. It is hoped that the evaluation of both treatments in ‘real-world’ settings will increase the generalizability of the study findings.

The study will also have three limitations. First, it may be difficult to generalize the results of the present study because of the setting and location where the present study is conducted. Considering the feasibility, the present study will be conducted in university hospitals and a National Center Hospital. Therefore, it should be cautious to generalize the results in the present study. Second, the finding cannot be generalized to patients with a BMI below 14. Future studies are needed to evaluate the efficacy of CBT-E for patients with a BMI below 14. Third, it would be difficult to identify which factors of CBT-E are efficacious in improving ED pathology and weight recovery in the trial. Even with these limitations, given that there is no evidence-based treatment for AN in Japan, it would be valuable to explore the efficacy of CBT-E as a treatment package in this trial. If the study demonstrates the efficacy of CBT-E, it will serve as a cornerstone for establishing standards for AN treatment. With the standards for treatment of AN available, more and more medical professionals will be able to provide the appropriate treatment, and more and more patients will be able to receive the adequate treatment. As a result, many cases will accumulate and that will enable the study to identify which factors of CBT-E are efficacious in improving ED pathology and weight recovery.

## Electronic supplementary material

Below is the link to the electronic supplementary material.

Supplementary Material 1

## Data Availability

We cannot share our data because sharing data is not permitted by our hospital or ethics committee.

## List of Abbreviations

AN-BP: Anorexia nervosa binging/purging type
AN-R: Anorexia nervosa restricting type
AN: Anorexia nervosa
BED: Binge eating disorder
BMI: Body mass index
BN: Bulimia nervosa
CIA: Clinical Impairment Assessment questionnaire
DSM-5: The Diagnostic and Statistical Manual of Mental Disorders, Fifth Edition
ED: Eating disorder
EDE-Q: The Eating Disorder Examination Questionnaire
HC: Healthy control
SCID-5 RV: The Structured Clinical Interview for DSM-5, Research Version

## References

[1] Treasure J, Duarte TA, Schmidt U (2020). Eating disorders. Lancet.

[2] Hay P, Chinn D, Forbes D, Madden S, Newton R, Sugenor L (2014). Royal Australian and New Zealand College of Psychiatrists clinical practice guidelines for the treatment of eating disorders. Aust N Z J Psychiatry.

[3] Dutch Foundation for Quality Development in Mental Healthcare (2017). Practice guideline for the treatment of eating disorders [Zorgstandaard Eetstoornissen].

[4] UK National Guideline Alliance. Eating disorders: recognition and treatment; 2017.

[5] Poulsen S, Lunn S, Daniel SI, Folke S, Mathiesen BB, Katznelson H (2014). A randomized controlled trial of psychoanalytic psychotherapy or cognitive-behavioral therapy for bulimia nervosa. Am J Psychiatry.

[6] Fairburn CG, Bailey-Straebler S, Basden S, Doll HA, Jones R, Murphy R (2015). A transdiagnostic comparison of enhanced cognitive behaviour therapy (CBT-E) and interpersonal psychotherapy in the treatment of eating disorders. Behav Res Ther.

[7] Wonderlich SA, Peterson CB, Smith TL, Klein M, Mitchell JE, Crow SJ et al. Integrative cognitive-affective therapy for bulimia nervosa. The treatment of eating disorders: A clinical handbook 2010:317 – 38.

[8] Dalle Grave R, Calugi S, Sartirana M, Fairburn CG (2015). Transdiagnostic cognitive behaviour therapy for adolescents with an eating disorder who are not underweight. Behav Res Ther.

[9] Fairburn CG, Cooper Z, Doll HA, O’Connor ME, Palmer RL, Dalle Grave R (2013). Enhanced cognitive behaviour therapy for adults with anorexia nervosa: a UK-Italy study. Behav Res Ther.

[10] Calugi S, Dalle Grave R, Sartirana M, Fairburn CG (2015). Time to restore body weight in adults and adolescents receiving cognitive behaviour therapy for anorexia nervosa. J Eat Disord.

[11] Dalle Grave R, Sartirana M, Calugi S (2019). Enhanced cognitive behavioral therapy for adolescents with anorexia nervosa: outcomes and predictors of change in a real-world setting. Int J Eat Disord.

[12] Solmi M, Wade TD, Byrne S, Del Giovane C, Fairburn CG, Ostinelli EG (2021). Comparative efficacy and acceptability of psychological interventions for the treatment of adult outpatients with anorexia nervosa: a systematic review and network meta-analysis. Lancet Psychiatry.

[13] Zipfel S, Wild B, Groß G, Friederich HC, Teufel M, Schellberg D (2014). Focal psychodynamic therapy, cognitive behaviour therapy, and optimised treatment as usual in outpatients with anorexia nervosa (ANTOP study): randomised controlled trial. Lancet.

[14] Byrne S, Wade T, Hay P, Touyz S, Fairburn CG, Treasure J (2017). A randomised controlled trial of three psychological treatments for anorexia nervosa. Psychol Med.

[15] Signorini DF, Leung O, Simes RJ, Beller E, Gebski VJ, Callaghan T (1993). Dynamic balanced randomization for clinical trials. Stat Med.

[16] Japan Society for Eating Disorders (Nihon Sessyoku Syougai Gakkai). Eating Disorders Treatment Guidelines (Sessyoku Syougai Tiryou Gaidorain). Igaku Shoin; 2012.

[17] Signorini R, Sheffield J, Rhodes N, Fleming C, Ward W (2018). The effectiveness of enhanced cognitive behavioural therapy (CBT-E): a naturalistic study within an out-patient eating disorder service. Behav Cogn Psychother.

[18] Wade S, Byrne S, Allen K (2017). Enhanced cognitive behavioral therapy for eating disorders adapted for a group setting. Int J Eat Disord.

[19] Fairburn CG (2008). Cognitive behavior therapy and eating disorders.

[20] Otani M, Hiraide M, Horie T, Mitsui T, Yoshida T, Takamiya S (2021). Psychometric properties of the eating disorder examination-questionnaire and psychopathology in japanese patients with eating disorders. Int J Eat Disord.

[21] Bohn K, Doll HA, Cooper Z, O’Connor M, Palmer RL, Fairburn CG (2008). The measurement of impairment due to eating disorder psychopathology. Behav Res Ther.

[22] Fairburn CG, Beglin SJ (1994). Assessment of eating disorders: interview or self-report questionnaire?. Int J Eat Disord.

[23] von Brachel R, Hotzel K, Hirschfeld G, Rieger E, Schmidt U, Kosfelder J (2014). Internet-based motivation program for women with eating disorders: eating disorder pathology and depressive mood predict dropout. J Med Internet Res.

[24] Horie T, Hiraide M, Takakura S, Hata T, Sudo N, Yoshiuchi K (2020). Development of a new japanese version of the clinical impairment Assessment Questionnaire. Biopsychosoc Med.

[25] The WHO. THE GLOBAL HEALTH OBSERVATORY Explore a world of health data, Prevalence of underweight among adults, BMI < 18.5 (crude estimate) (%). 2022. www.who.int/data/gho/data/indicators/indicator-details/GHO/prevalence-of-underweight-among-adults-bmi-18-(crude-estimate)-(). Accessed Jan 10,2023 2023.

[26] Harada T, Yamauchi T, Miyawaki D, Miyamoto S, Yoshida H, Nishimoto K (2021). Anorexia nervosa restricting type has increased in severity over three decades: japanese clinical samples from 1988 to 2018. Int J Eat Disord.

