# Supplementary material for: A multi-center, randomized, parallel-group study to compare the efficacy of enhanced cognitive behavior therapy (CBT-E) with treatment as usual (TAU) for anorexia nervosa: study protocol
Source: Biopsychosoc Med. 2023 May 29;17:20. doi: 10.1186/s13030-023-00277-2 (PMC10226199; doi:10.1186/s13030-023-00277-2)
Supplement: Supplementary file 1 — Supplementary Material 1 [file 13030_2023_277_MOESM1_ESM.docx]

Table S1. Session frequency by BMI category

|  | 14 ≤ BMI < 16  **40 sessions** | | 16.0 ≤ BMI ≤ 17.5  **30 sessions** | | **17.5 < BMI < 18.5**  **25 sessions** | |
| --- | --- | --- | --- | --- | --- | --- |
|  | Frequency | Sessions | Frequency | Sessions | Frequency | Sessions |
| Month 1 | 2 sessions  a week | 8 | 2 sessions  a week | 8 | 2 sessions a week for week 1, 1 session a week for week 2-4 | 5 |
| Month 2 | 1 session  a week | 12 | 1 session  a week | 12 | 1 session  a week | 9 |
| Month 3 | 1 session  a week | 16 | 1 session  a week | 16 | 1 session  a week | 13 |
| Month 4 | 1 session  a week | 20 | 1 session  a week | 20 | 1 session  a week | 17 |
| Month 5 | 1session  a week | 24 | Two sessions  a month | 22 | Two sessions a month | 19 |
| Month 6 | 1 session  a week | 28 | Two sessions  a month | 24 | 1 session  every 3 weeks | 20 |
| Month 7 | 1 session  a week | 32 | Two sessions  a month | 26 | 1 session  every 3 weeks | 22 |
| Month 8 | 1 session  a week | 36 | 1 session  every 3 weeks | 27 | 1 session  every 3 weeks | 23 |
| Month 9 | 2 sessions  a month | 38 | 1 session every 3 weeks | 29 | 1 session  every 3 weeks | 24 |
| Month 10 | 2 sessions  a month | 40 | 1 session every 3 weeks | 30 | 1 session  every 3 weeks | 25 |

Table S2. The PICO of the study.

| Patients: | AN-R or AN-BP who have a BMI between 14.0 and 18.5 |
| --- | --- |
| Interventions: | CBT-E |
| Comparisons: | TAU |
| Outcome: | BMI, EDE-Q, CIA |

Table S3. The FINER of the study.

| Feasibility: | Five to ten new patients are diagnosed with AN in outpatient settings per month solely in The University of Tokyo hospital, 12 to 24 participants will be incorporated in this trial per year even if 20% of the patients participate in the trial. Human resources are adequate to provide treatment for research. We have eight experienced physicians who have more than two years of experience in treating eating disorders. Funding is provided by the Ministry of Health, Labor and Welfare. |
| --- | --- |
| Interesting: | There are no cohort or randomized controlled trials on the efficacy of CBT-E for patients with AN in Japan. Only two papers have been published to date that evaluated the efficacy of CBT-E in randomized controlled trials. |
| Novelty: | There are no studies on the efficacy of CBT-E for patients with AN in Japan (in non-western countries), and no other psychotherapies has been proven to be efficacious. |
| Ethical: | Ethical approval for the study has been obtained from the central review board of the ethics committees of the Graduate School of Medicine of the University of Tokyo. All researchers and collaborators in this study have no competing interests. Similar trials have already been conducted in the U.S. and Europe and no severe adverse effects have never been reported in those trials. |
| Relevant: | There is no standard treatment of patients with AN in Japan. If the study demonstrates the efficacy of CBT-E, it will serve as cornerstone for establishing standards for AN treatment. With the standards for treatment of AN available, more and more medical professionals can provide the appropriate treatment, and more and more patients can receive the adequate treatment. Therefore, a greater number of cases will accumulate and that will enable the study to identify which factors of CBT-E are efficacious in improving ED pathology and weight recovery. |
